# Clinical Outcomes and Prognostic Implications of TAVR in Patients With Active Cancer: A Meta‐Analysis

**DOI:** 10.1002/clc.70121

**Published:** 2025-03-24

**Authors:** Parsa Saberian, Rafael Contreras, Anoop Gurram, Amir Nasrollahizadeh, Narsimha Rao Keetha, Anthony Loc Nguyen, Sandeep Samethadka Nayak, Mohammad‐Hossein Keivanlou, Mohammad Hashemi, Ehsan Amini‐Salehi, Daniyal Ameen

**Affiliations:** ^1^ Cardiovascular Research Center Hormozgan University of Medical Sciences Bandar Abbas Iran; ^2^ Department of Internal Medicine Yale New Heaven Health Bridgeport Hospital 267 Grant St Bridgeport CT USA; ^3^ Department of Hospital Medicine Cleveland Clinic Cleveland OH USA; ^4^ Tehran Heart Center, Cardiovascular Diseases Research Institute Tehran University of Medical Sciences Tehran Iran; ^5^ Ohio Kidney and Hypertension Center 7255 Middleburg Hts OH USA; ^6^ Division of Hematology/Oncology, Department of Internal Medicine UC San Diego Health Moores Cancer Center San Diego CA USA; ^7^ School of Medicine Guilan University of Medical Sciences Rasht Iran

**Keywords:** cancer, complications, meta‐analysis, mortality, prognosis, TAVR

## Abstract

**Background:**

Transcatheter aortic valve replacement (TAVR) is an alternative to surgical aortic valve replacement (SAVR) for high‐risk patients with severe aortic stenosis (AS). However, the clinical outcomes and prognostic implications of TAVR in patients with active cancer remain uncertain. This meta‐analysis evaluates procedural success, complications, and survival outcomes of TAVR in patients with and without active cancer.

**Methods:**

A comprehensive literature search was conducted across PubMed, Scopus, and Web of Science databases. Statistical analysis was performed using a random‐effects model. Statistical analyses were conducted using STATA version 18.0.

**Results:**

The results of the meta‐analysis showed no significant difference in in‐hospital mortality between cancer and non‐cancer patients (OR = 1.17; 95% CI: 0.83, 1.65; *p* = 0.27). Similarly, 30‐day mortality did not differ between the two groups (OR = 0.93; 95% CI: 0.72, 1.19; *p* = 0.49). However, 1‐year mortality was significantly higher in cancer patients (OR = 1.93; 95% CI: 1.45, 2.56; *p* < 0.01). Two‐year mortality was also higher in cancer patients (OR = 2.65; 95% CI: 1.79, 3.93; *p* < 0.01). No significant differences were observed in major bleeding, acute kidney injury, stroke, or permanent pacemaker implantation between the groups.

**Conclusion:**

While TAVR offers comparable in‐hospital and short‐term survival outcomes for cancer and non‐cancer patients, long‐term mortality is significantly higher in those with active cancer. These findings suggest that TAVR is a viable option for cancer patients with severe AS but requires careful long‐term prognostic considerations. Further studies are needed to optimize management strategies for this complex population.

## Graphical abstract

Clinical outcomes of transcatheter aortic valve replacement (TAVR) in patients with active cancer compared to those without cancer.

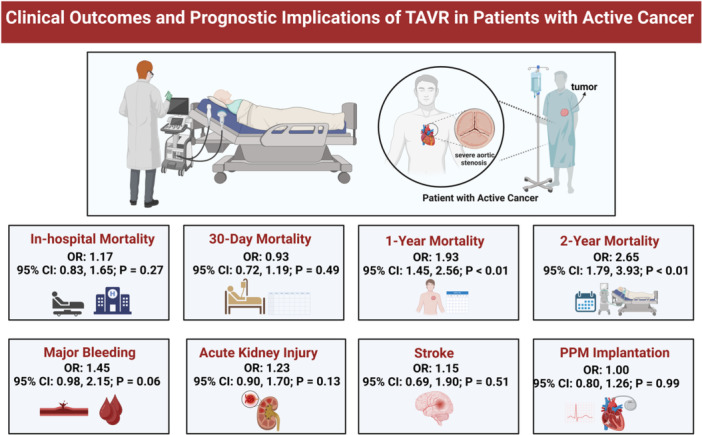

**Abbreviations**
ACC/AHA: American College of Cardiology/American Heart Association
AKI: Acute Kidney Injury
AS: Aortic Stenosis
CI: Confidence Interval
CVD: Cardiovascular Disease
EACTS: European Association for Cardio‐Thoracic Surgery
ESC: European Society of Cardiology
EuroSCORE: European System for Cardiac Operative Risk Evaluation
JBI: Joanna Briggs Institute
OR: Odds Ratio
PPM: Permanent Pacemaker
PRISMA: Preferred Reporting Items for Systematic Reviews and Meta‐Analyses
PROSPERO: International Prospective Register of Systematic Reviews
SAVR: Surgical Aortic Valve Replacement
SMD: Standardized Mean Difference
STS: Society of Thoracic Surgeons (Score)
TAVR: Transcatheter Aortic Valve Replacement

## 1 Introduction

Transcatheter aortic valve replacement (TAVR) has emerged as a transformative treatment modality for patients with severe aortic stenosis (AS) who are deemed high‐risk or inoperable for surgical aortic valve replacement (SAVR) [1, 2, 3]. The minimally invasive nature of TAVR offers an attractive alternative for patients with significant comorbidities, including those with active cancer [4, 5, 6, 7]. This specific subgroup of patients presents unique challenges and considerations, given their complex clinical profiles and competing risks associated with both cancer and cardiovascular disease (CVD) [8, 9, 10, 11]. The interplay between cancer‐related systemic inflammation, hypercoagulability, and the potential impact of antithrombotic therapy poses additional problems in the management of these patients [12, 13, 14, 15].

The prevalence of AS in cancer patients is expected to rise due to shared risk factors such as advanced age and systemic inflammation, as well as the cardiotoxic effects of certain cancer treatments [16, 17, 18, 19]. Despite the growing number of patients with coexistent cancer and severe AS, the optimal management of this population is challenging [18, 20].

Recent studies have begun to explore the feasibility of TAVR in patients with active cancer, highlighting potential benefits and complications in these individuals. However, these findings are often limited by small sample sizes, heterogeneous patient populations, and variable cancer stages [21, 22, 23]. This meta‐analysis aims to address these gaps by systematically evaluating the clinical outcomes of TAVR in patients with active cancer. By synthesizing data from existing studies, this review seeks to provide a comprehensive assessment of procedural success rates, perioperative complications, and survival outcomes.

## 2 Methods

This meta‐analysis adhered to the Preferred Reporting Items for Systematic Reviews and Meta‐Analyses (PRISMA) guidelines and was registered in the International Prospective Register of Systematic Reviews (PROSPERO; Registration ID: CRD42025634229) [24]. The aim was to evaluate the clinical outcomes and prognostic implications of TAVR in patients with active cancer.

### 2.1 Search Strategy

A comprehensive literature search was conducted across PubMed, Scopus, and Web of Science databases from their inception to December 1, 2024. The search strategy utilized a combination of Medical Subject Headings (MeSH) and free‐text terms, including “TAVR,” “transcatheter valve replacement,” “cancer,” and “neoplasm.” No language restrictions were applied. Additional relevant articles were identified by screening the references of included studies. The detailed search formula for each database is provided in Table S1.

### 2.2 Study Selection and Eligibility Criteria

Studies were included if they: (1) compared outcomes of TAVR in patients with active cancer to those without cancer, (2) provided quantitative data on clinical outcomes such as mortality, procedural success, or complications, and (3) involved human subjects aged 18 years or older. Excluded were case reports, reviews, editorials, and studies with insufficient data for analysis.

Two independent reviewers screened titles and abstracts for relevance, followed by full‐text assessments to confirm eligibility. Discrepancies were resolved through discussion or consultation with a third reviewer.

### 2.3 Quality Assessment

The quality assessment of the studies included was performed independently by two researchers using the Joanna Briggs Institute (JBI) Critical Appraisal Checklist [25, 26, 27]. Any disagreements between the researchers were resolved with the help of a third researcher to reach the final conclusion.

### 2.4 Data Extraction

Two reviewers independently extracted data using a standardized form. Extracted variables included study characteristics (author, year, location, study design), patient demographics, cancer type and stage, procedural details, and clinical outcomes (e.g., in‐hospital mortality, 30‐day mortality, stroke, myocardial infarction, major bleeding). Where data were missing, corresponding authors were contacted.

### 2.5 Statistical Analyses

A random‐effects model was used to pool outcome measures, accounting for heterogeneity across studies. Effect sizes for binary outcomes were expressed as odds ratios (ORs) with 95% confidence intervals (CIs). Heterogeneity was assessed using the I² statistic, with values above 50% indicating substantial heterogeneity. Sensitivity analyses were conducted to examine the robustness of pooled estimates by excluding one study at a time. Publication bias was evaluated using funnel plots, Egger's test, and the trim‐and‐fill method. Statistical analyses were performed using STATA version 18.0.

## 3 Results

### 3.1 Study Selection

**Figure 1:**
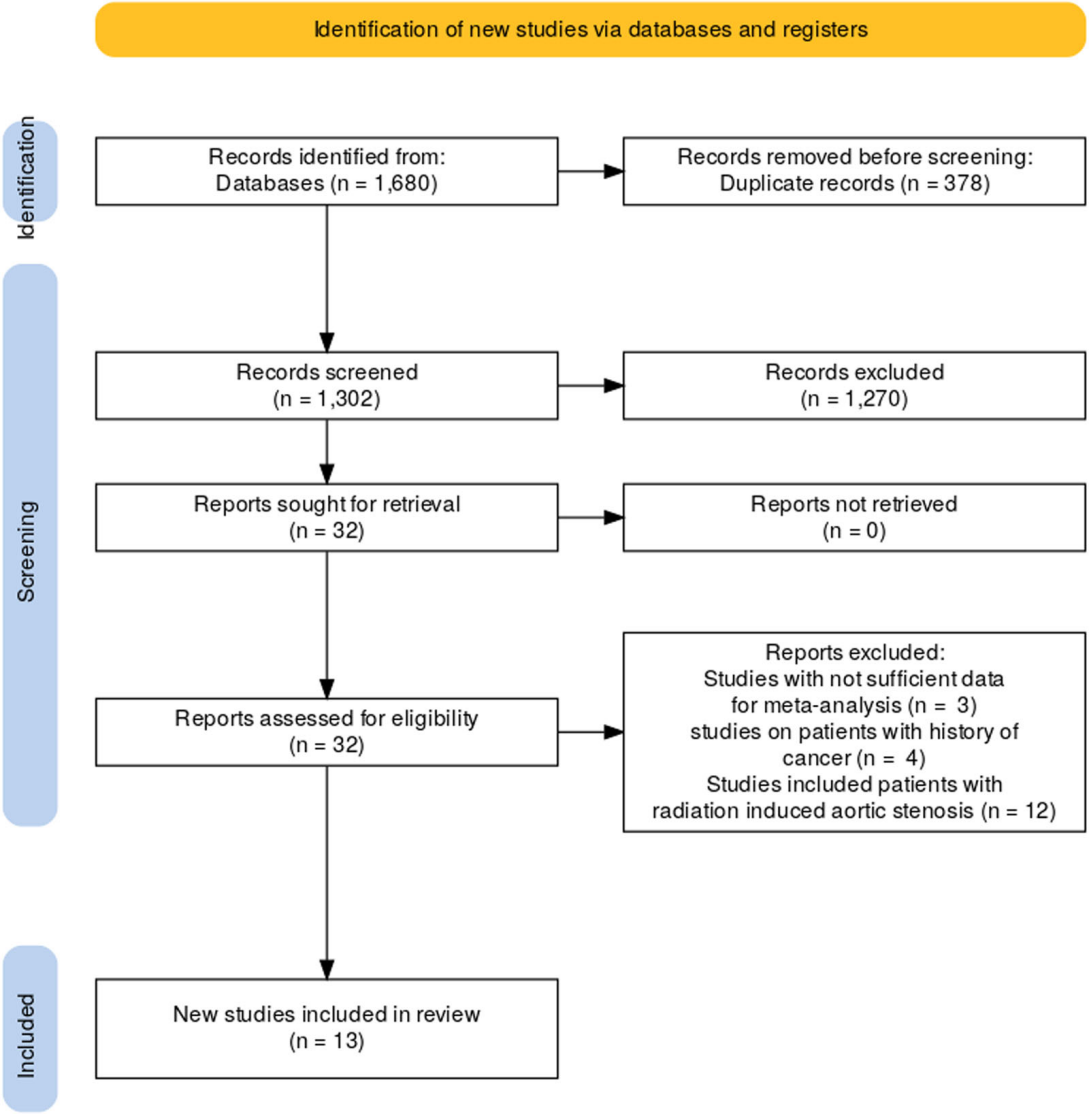
Study selection process.

A total of 1680 records were identified from databases. After screening titles and abstracts, 1270 records were excluded. The remaining 32 studies underwent full‐text assessment, during which 19 studies were excluded at this stage. Ultimately, 13 studies were included in the review (Figure 1).

### 3.2 Study Characteristics

**Figure 2:**
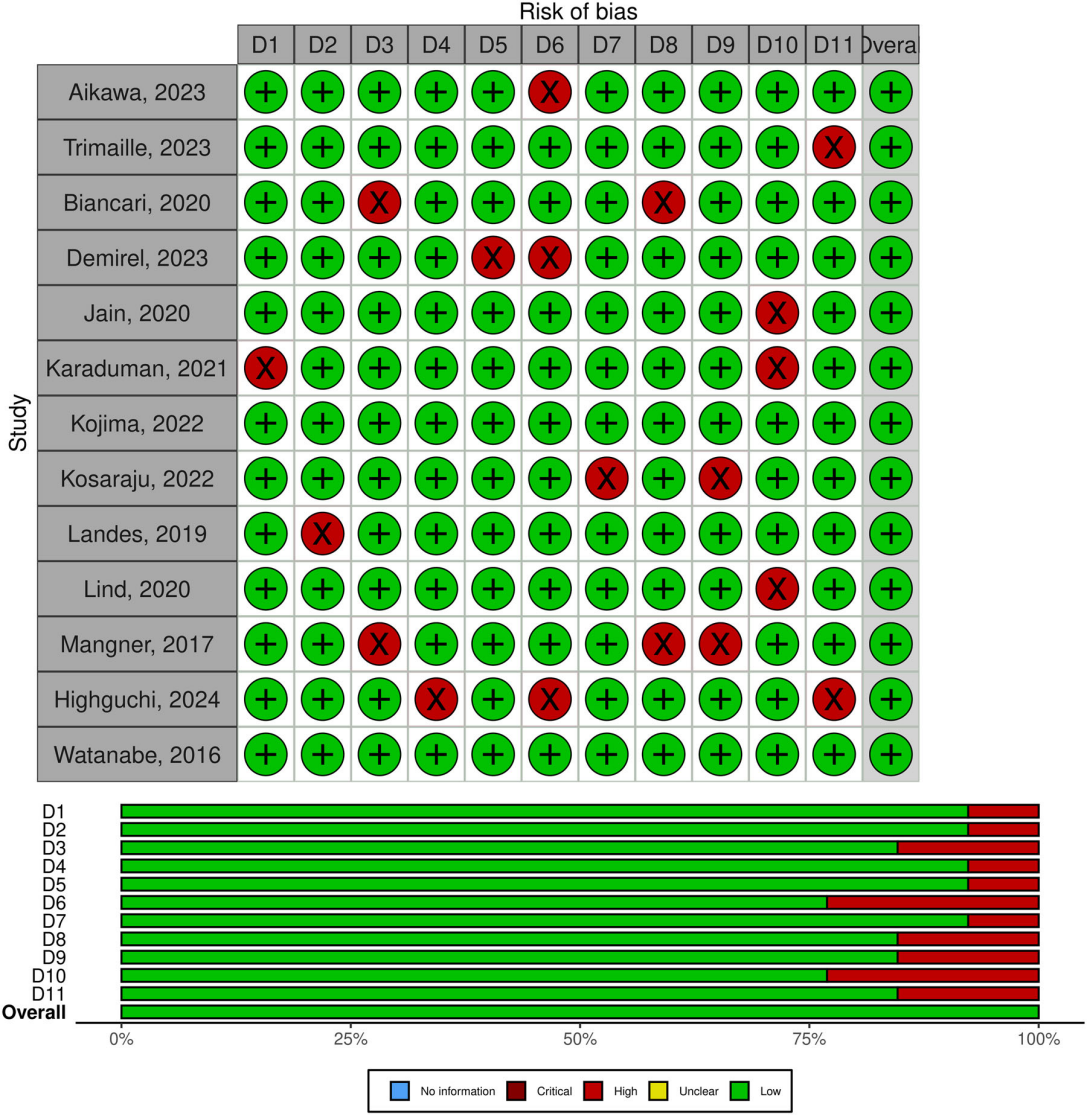
Quality of included studies bases on JBI criteria.

This meta‐analysis included 13 studies published between 2016 and 2024, analyzing data collected over a period spanning from 2006 to 2024. These studies assessed the clinical outcomes of patients undergoing TAVR with active cancer compared to those without cancer. The majority of the studies were conducted in the United States [6, 28, 29] and Japan [22, 30, 31]. Germany ranked next, contributing two studies [32, 33]. Additional studies originated from France [34], Finland [35], Switzerland [36], and Turkey [37]. One study was conducted at a global level, encompassing data from multiple regions (Table 1). All included studies were cohort designs. The median follow‐up periods reported by the studies ranged from 1 to 60 months (Table 1). All the included studies were of high quality (Figure 2).

**Table 1:** Characteristics of included studies.

First author	Year of publication	Time set	Study design	Mean age (Cancer/Without Cancer)	Gender distribution (M/F)	The country	Population (Cancer/Without Cancer)	Population active cancer	STS score (Cancer/Without Cancer)^1^	Euro score (Cancer/Without Cancer)^2^	In hospital stroke (Case/Control)	In hospital AKI (Case/Control)	In hospital PPM implantation (Case/Control)	Major bleeding (Case/Control)
Aikawa et al. [6]	2023	2012–2019	Retrospective Cohort	79.6 ± 8.6/ 79.6 ± 8.4	56907/65666	USA	122573 (8013/114560)	8013	N. A	N. A	48/458	1330/14090	881/11341	1129/9508
Trimaille et al. [34]	2023	2010–2019	Cohort	82.8 (80.9 ± 7.3/83.0 ± 7.0)	611/514	France	1125 (88/1037)	88	6.9 ± 6.4/6.5 ± 5.8	4.9 ± 3.9/6.0 ± 7.0	N. A	N. A	N. A	26/192
Biancari et al. [35]	2020	2008–2017	Retrospective Cohort	(80.6 ± 6.6/81.4 ± 6.6)	1172/958	Finland	2130 (417/1713)	113	N. A	N. A	N. A	N. A	N. A	N. A
Demirel et al. [36]	2023	2015–2016	Retrospective Cohort	(81.0 ± 6.6/81.6 ± 6.7)	289/286	Switzerland	575 (26/449)	26	N. A	N. A	N. A	N. A	N. A	N. A
Jain et al. [28]	2020	2012–2015	Retrospective Cohort	N. A	30055/33297	USA	63352 (2849/60503)	2849	N. A	N. A	58/1438	509/9826	291/6380	709/12830
Karaduman et al. [37]	2021	2011–2019	Retrospective Cohort	77.6 ± 7.9 (74.6 ± 6.5/77.8 ± 8.0)	302/248	Turkey	550 (36/514)	10	N. A	7.4 ± 4.9/9.1 ± 5.8	N. A	N. A	N. A	2/16
Kojima et al. [22]	2022	2010–2019	Retrospective Cohort	N.A.	764/350	Japan	1114 (62/1052)	62	5.2 (3.4–7.3)/5.8 (3.9–8.2)	3.9 (2.2–7.3)/4.3 (2.7–6.8)	1/6	N.A.	2/96	1/24
Kosaraju et al. [29]	2022	2012–2020	Retrospective Cohort	80.8 ± 9.7/79.7 ± 10.9	246/309	USA	555 (107/448)	107	6.5 ± 5.0/6.4 ± 4.1	N. A	N. A	N. A	N. A	N. A
Landes et al. [38]	2019	2008–2016	Retrospective Cohort	78.8 ± 7.5/81.3 ± 7.1	1471/1273	Global	2744 (222/2522)	222	4.9 ± 3.4/6.2 ± 4.4	4.2 ± 3.2/5.4 ± 4.4	N. A	N. A	N. A	19/71
Lind et al. [32]	2020	2006–2018	Retrospective Cohort	81.4 ± 5.4/78.5 ± 6.4	487/405	Germany	892 (53/839)	53	5.4 (3.3–6)/6.0 (3.5–6.8)	N. A	3/28	10/158	5/133	2/51
Mangner et al. [33]	2017	2006–2014	Retrospective Cohort	81 (77–84)/81 (77–84)	883/687	Germany	1570 (99/1471)	99	6.0 (3.8–10.9)/6.7 (4.1–10.6)	N. A	N. A	N. A	N. A	N. A
Noguchi et al. [30]	2024	2013–2017	Cohort	82.1 ± 5.4/84.5 ± 5.2	1607/729	Japan	2336 (89/2247)	89	6.0 ± 4.0/8.3 ± 7.0	3.8 ± 3.3/5.6 ± 6.7	0/49	0/48	7/192	17/344
Watanabe et al. [31]	2016	2013–2015	Retrospective Cohort	83 (80–87)/85 (82–88)	496/253	Japan	749 (47/702)	47	N. A	3.1 (1.5–4.7)/3.9 (2.0–5.8)	0/18	N. A	1/38	12/248

Abbreviations: AKI, Acute Kidney Injury; EuroSCORE, European System for Cardiac Operative Risk Evaluation; PPM, Permanent Pacemaker; STS Score, Society of Thoracic Surgeons Score.

## 4 Results of Meta‐Analysis

### 4.1 In‐Hospital Mortality

**Figure 3:**
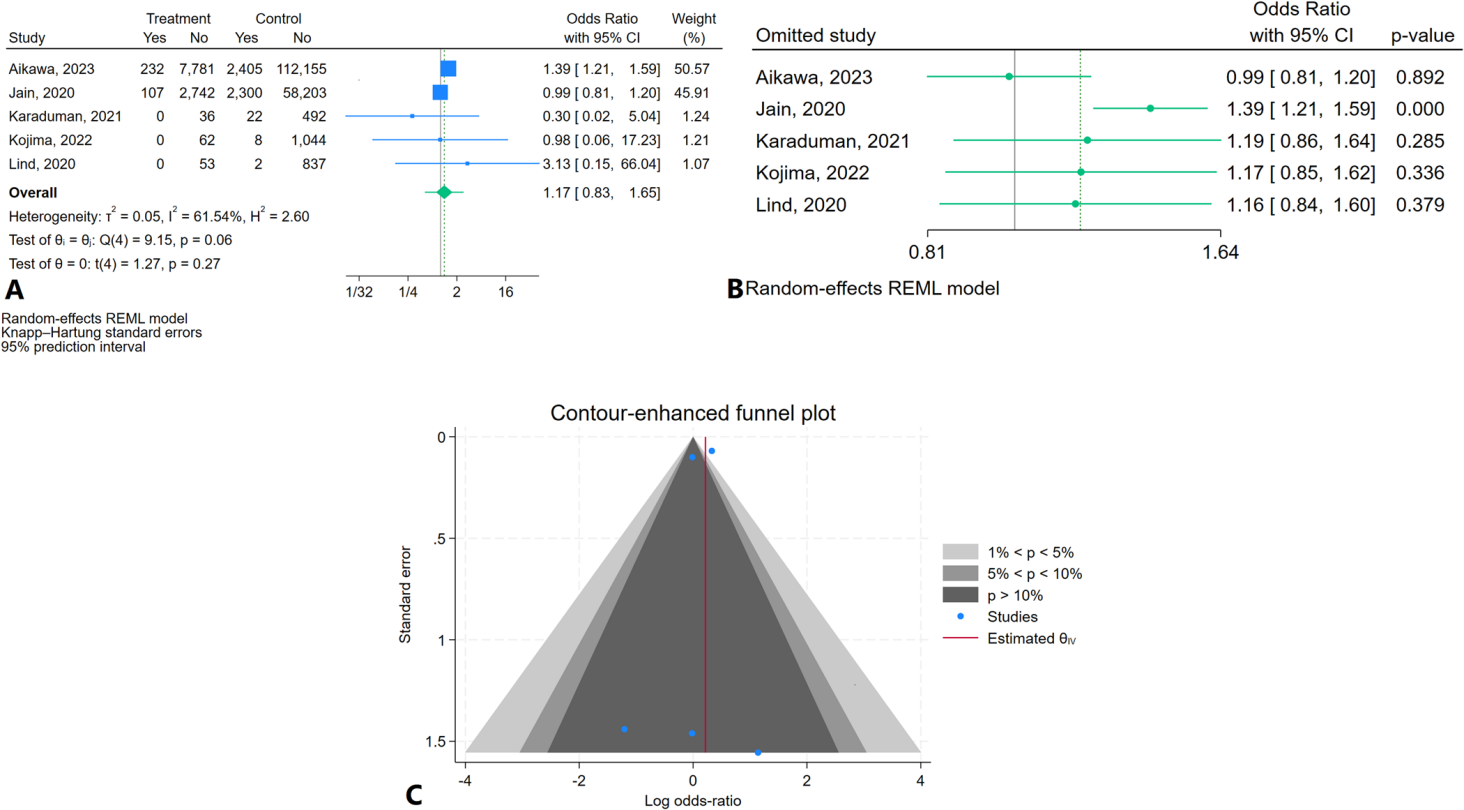
In‐hospital mortality in patients with active cancer compared to healthy individuals (A) Forest plot (B) Sensitivity analysis (C) Contour‐enhanced funnel plot.

The results of the study revealed no significant difference in in‐hospital mortality between patients with active cancer undergoing TAVR and those without cancer (OR = 1.17; 95% CI: 0.83, 1.65, *p* = 0.27). The 95% prediction interval was (0.49, 2.77) (Figure 3A). Heterogeneity across the included studies was moderate, with an I² value of 61.54% (*p* = 0.06). Sensitivity analysis indicated a higher in‐hospital mortality in patients with active cancer who underwent TAVR compared to individuals without cancer after the removal of the Jain 2020 study (OR = 1.39; 95% CI: 1.21, 1.59, *p* < 0.01) (Figure 3B). Regarding publication bias, the contour‐enhanced funnel plot showed some asymmetry, particularly with smaller studies. However, Egger's test (*p* = 0.75) and Begg's test (*p* = 0.80) did not indicate significant publication bias (Figure 3C).

### 4.2 30‐day Mortality

**Figure 4:**
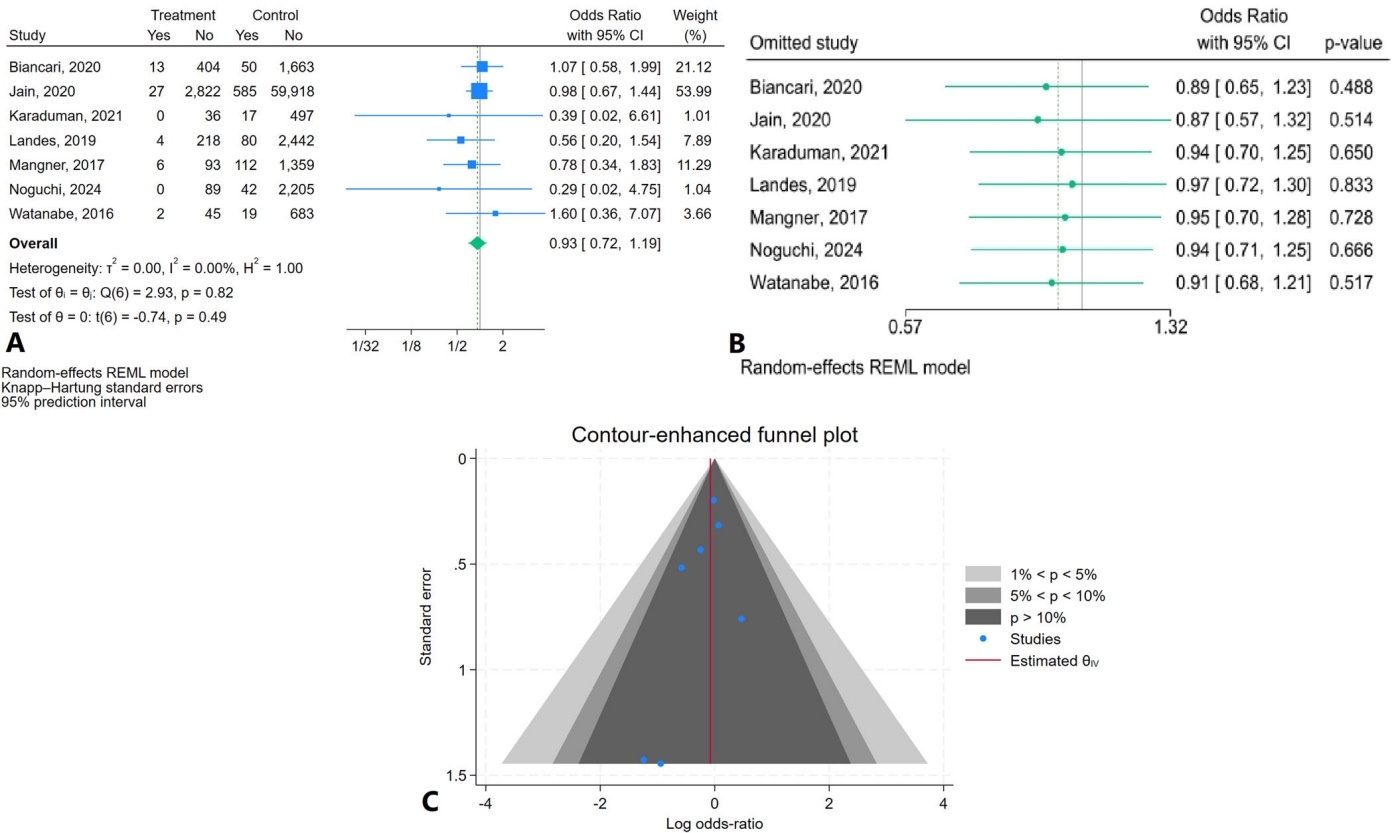
30‐day mortality in patients with active cancer compared to healthy individuals (A) Forest plot (B) Sensitivity analysis (C) Contour‐enhanced funnel plot.

The results of the study revealed no significant difference in 30‐day mortality between patients with active cancer undergoing TAVR and those without cancer (OR = 0.93; 95% CI: 0.72, 1.19, *p* = 0.49). The 95% prediction interval was (0.63, 1.34) (Figure 4A). Heterogeneity across the included studies was low, with an I² value of 0.00% (*p* = 0.82), indicating that the results were consistent across studies. Sensitivity analysis showed that omitting any single study did not significantly affect the overall odds ratio, as no individual study had a substantial impact on the pooled estimate (Figure 4B). Regarding publication bias, the contour‐enhanced funnel plot showed symmetry, suggesting no major bias (Figure 4C). Both Egger's test (*p* = 0.36) and Begg's test (*p* = 0.54) further confirmed the absence of significant publication bias.

### 4.3 1‐Year Mortality

Patients with active cancer had significantly higher 1‐year mortality compared to healthy individuals undergoing TAVR (OR = 1.93; 95% CI: 1.45, 2.56, *p* < 0.01). The 95% prediction interval was (1.33, 2.78) (Figure S1A). Heterogeneity across the included studies was minimal, with an I² value of 0.00% (*p* = 0.53). Sensitivity analysis showed no significant change in the pooled odds ratio after omitting any individual study (Figure S1B). The contour‐enhanced funnel plot suggested the presence of publication bias (Figure S1C). However, Egger's test (*p* = 0.77) and Begg's test (*p* = 0.80) did not indicate significant publication bias.

### 4.4 In Hospital Acute Kidney Injury (AKI)

There was no significant difference in in‐hospital AKI between patients with active cancer undergoing TAVR and healthy individuals (OR = 1.23; 95% CI: 0.90, 1.70, *p* = 0.13). The 95% prediction interval was (0.55, 2.74) (Figure S2A). Heterogeneity across the included studies was high, with an I² value of 81.85% (*p* < 0.01). Sensitivity analysis showed higher odds of in‐hospital AKI after the removal of Aikawa, 2023 (OR = 1.12; 95% CI: 1.01, 2.23, *p* = 0.02), Jain 2020 (OR = 1.35; 95% CI: 1.07, 1.71, *p* = 0.01), and Noguchi, 2020 (OR = 1.24; 95% CI: 1.01, 1.53, *p* = 0.03) (Figure S2B). Regarding publication bias, the contour‐enhanced funnel plot showed no indication of publication bias (Figure S2C), confirmed by Egger's test (*p* = 0.19) and Begg's test (*p* = 0.73).

### 4.5 In Hospital Permanent Pacemaker (PPM) Implantation

There was no significant difference in in‐hospital PPM implantation between patients with active cancer undergoing TAVR and healthy individuals (OR = 1.00; 95% CI: 0.80, 1.26, *p* = 0.99). The 95% prediction interval was (0.67, 1.48) (Figure S3A). Heterogeneity across the included studies was substantial, with an I² value of 50.13% (*p* = 0.07). Sensitivity analysis showed no significant change in the pooled odds ratio after omitting any individual study (Figure S3B). Regarding publication bias, the contour‐enhanced funnel plot suggested no significant publication bias (Figure S3C), although Egger's test (*p* = 0.02) indicated significant publication bias, while Begg's test (*p* = 0.70) was not significant.

### 4.6 Major Bleeding

There was no significant difference in major bleeding between patients with active cancer undergoing TAVR and healthy individuals (OR = 1.45; 95% CI: 0.98, 2.15, *p* = 0.06). The 95% prediction interval was (0.52, 3.98) (Figure S4A). Heterogeneity across the included studies was substantial, with an I² value of 93.29% (*p* < 0.01). Sensitivity analysis showed a higher risk of bleeding in patients with active cancer after the removal of Kojima, 2022 (OR = 1.48; 95% CI: 1.05, 2.08, *p* = 0.02), Landes, 2019 (OR = 1.33; 95% CI: 1.00, 1.76, *p* = 0.04), Lind, 2020 (OR = 1.51; 95% CI: 1.07, 2.12, *p* = 0.01), and Watanabe, 2016 (OR = 1.62; 95% CI: 1.22, 2.15, *p* < 0.01) (Figure S4B). Regarding publication bias, the contour‐enhanced funnel plot suggested some asymmetry (Figure S4C). However, neither Egger's test (*p* = 0.35) nor Begg's test (*p* = 0.46) indicated significant publication bias.

### 4.7 In‐Hospital Stroke

There was no significant difference in in‐hospital stroke between patients with active cancer undergoing TAVR and healthy individuals (OR = 1.15; 95% CI: 0.69, 1.90, *p* = 0.51). The 95% prediction interval was (0.39, 3.31) (Figure S5A). Heterogeneity across the included studies was moderate, with an I² value of 51.57% (*p* = 0.06). Sensitivity analysis showed a higher risk of in‐hospital stroke in patients with active cancer after the removal of Jain 2020 (OR = 1.48; 95% CI: 1.12, 1.97, *p* < 0.01) (Figure S5B). Regarding publication bias, the contour‐enhanced funnel plot suggested no significant publication bias (Figure S5C), confirmed by Egger's test (*p* = 0.74) and Begg's test (*P* = 1.00).

### 4.8 Two‐Year Mortality and One‐Year CVD After Procedure

Patients with active cancer undergoing TAVR had significantly higher 2‐year mortality compared to healthy individuals (OR = 2.65; 95% CI: 1.79, 3.93, *p* < 0.01) (Figure S6A). However, there was no significant difference regarding 1‐year CVD (OR = 1.01; 95% CI: 0.68, 1.49, *p* = 0.97) (Figure S6B). Only two studies were available for these outcomes.

## 5 Discussion

Cardiovascular diseases and malignancies share numerous common risk factors and are known as leading causes of mortality and morbidity worldwide [39]. The concomitant presentation of valvular heart disease, specifically aortic valve pathology, and cancer is not rare, and it requires rigorous and precise diagnostic and therapeutic approaches [18, 40]. The European Society of Cardiology (ESC) guidelines and Journal of the American College of Cardiology (JACC) state‐of‐the‐art review suggest a tailored, individualized approach for managing cardiac interventions in candidates with active malignancies [41, 42]. Due to significant advancements in device technologies, TAVR has become a favorable approach for managing aortic valve pathology, specifically in patients with intermediate and high surgical risk [43]. Herein, we comprehensively evaluate the clinical and safety outcomes of TAVR in patients with active cancer, an important population underrepresented in clinical trials, compared to non‐cancer controls. Regarding in‐hospital outcomes, mortality, AKI, PPM implantation, and stroke were comparable between the two groups. Furthermore, TAVR resulted in similar rates of 30‐day mortality, 1‐year CVD, and major bleeding in both patients with and without active cancer. However, patients with active malignancies experienced significantly higher rates of 1‐ and 2‐year mortality.

Several meta‐analyses have been conducted to explore the efficacy and safety of TAVR in cancer patients [44, 45, 46]. These meta‐analyses reported similar rates of short‐term mortality and complications in cancer patients (active or previous) compared to controls. In addition, the significantly higher rate of long‐term mortality was consistent across studies [44, 45, 46].

However, candidates with active cancer are a distinct group of patients from those with a history of malignancies [47]. Moreover, the comprehensive knowledge regarding timing, indications, and considerations of transcatheter interventions in patients with active cancer is not fully established [48]. Felix et al. aimed to comprehensively evaluate the efficacy and safety of TAVR exclusively in patients with active cancer (not cured) [49]. They found that patients with active cancer have higher short‐ and long‐term mortality compared to those without cancer. The discrepancy between our short‐term mortality results may be attributable to the different number of included studies and the definition of short‐term mortality in Felix's study, which combined in‐hospital and 30‐day mortality. While the higher rate of long‐term mortality in cancer patients was consistently reported across all studies, it is noteworthy that the rate of 1‐year cardiovascular diseases was comparable between the two groups. This suggests that the high rate of all‐cause mortality can be related to non‐cardiovascular factors, potentially the severity and progression of the underlying cancer.

In terms of bleeding, consistent with previous studies, our analysis revealed similar outcomes between the two groups [45, 46, 49, 50]. However, Felix et al. reported a significantly higher rate of bleeding in patients with active cancer [49]. Furthermore, the borderline significance of bleeding (*p* = 0.06) warrants further evaluation. Patients with active cancer are at high risk of hemostasis dysregulation. Platelet dysfunction, increased risk of thromboembolic events, and the potential use of anticoagulants complicate the balance between thrombosis and bleeding [51, 52, 53]. The inconsistency across studies necessitates concise and individualized attention in this regard.

Regarding pacemaker implementation, in contrast to our findings, Bendary et al. reported significantly higher rates of pacemaker need after TAVR in cancer patients [50]. It is noteworthy that, regarding the need for a pacemaker, Bendary et al. pooled the data from only three studies, while we pooled results from six studies. However, due to the arrhythmogenic effects of some chemotherapy drugs, tailored follow‐up of active cancer patients is warranted [54].

It is important to highlight that some heterogeneity was detected in the sensitivity analyses of our meta‐analysis, possibly indicating that there was heterogeneity in cancer type, stage, or treatment regimen across different studies, particularly for AKI and bleeding outcomes.

While our study primarily focused on the impact of TAVR in patients with active cancer, previous research has also explored the effectiveness of TAVR in individuals with a history of cancer. For example, a meta‐analysis by Murphy et al. investigated TAVR outcomes in patients with a history of cancer, both perioperatively and long‐term [55]. Their findings indicated that short‐term mortality and long‐term all‐cause mortality did not significantly differ between patients with and without a history of cancer. In addition, there were no notable differences in periprocedural complications such as stroke, bleeding, AKI, or pacemaker implantation, suggesting that patients with a cancer history may experience similar outcomes to non‐cancer patients.

Similarly, Yasmin et al. conducted a meta‐analysis to assess TAVR outcomes in cancer survivors who had undergone chest radiation therapy (C‐XRT) [56]. They found that TAVR outcomes, including all‐cause mortality, efficacy, and safety, were comparable between cancer survivors with and without prior C‐XRT, except for a higher incidence of worsening congestive heart failure (CHF) in those with a history of C‐XRT. This further suggests that, for cancer survivors, prior cancer treatments like C‐XRT do not significantly alter TAVR effectiveness or safety, aside from specific complications such as CHF.

These studies highlight that while the outcomes for patients with a history of cancer may mirror those of non‐cancer patients, the situation is different for patients with active cancer. Active cancer and ongoing treatments like chemotherapy can pose additional risks, potentially influencing procedural success and long‐term survival.

An important issue that arises from the current studies is the variability in approaches and cancer types, which impact outcomes after TAVR. For example, Sakai et al. presented a case report involving two patients with severe AS and lung cancer, who successfully underwent TAVI and lung resection without any complications [57]. However, since their study was a case report, the results cannot be generalized to the broader population of cancer patients undergoing TAVR. This highlights the challenge in forming firm conclusions about the treatment of cancer patients with AS based on limited case studies.

In contrast, Aikawa et al. evaluated outcomes of TAVR in patients with active cancer and investigated the impact of cancer types, including metastatic disease. While they found that active cancer was not associated with increased in‐hospital mortality after adjusting for potential confounders, they did observe an increased risk of readmission at 30, 90, and 180 days post‐TAVR. In addition, patients with active cancer had a higher likelihood of requiring transfusion due to bleeding at 30 days. The study also highlighted that specific cancer types, such as active colon cancer and metastatic cancer, were particularly associated with these complications. Notably, colon cancer, prostate cancer, and any metastatic cancer increased the risk of bleeding at 30 days post‐TAVR, further complicating the management of these patients [5].

Furthermore, Kojima et al. found that patients with active cancer and metastasis had worse survival rates compared to those without metastasis. This finding underscores the importance of considering the presence of metastasis when evaluating cancer patients for TAVR, as it can have a significant impact on both short‐ and long‐term survival outcomes [22].

Given the gaps in the existing literature, our meta‐analysis was limited by the insufficient number of studies available for robust conclusions. This highlights the need for future studies that provide more comprehensive and coherent data on cancer types, stages, metastatic status, treatment regimens, and the type of TAVR procedures used. Such research will be crucial in understanding how these factors influence TAVR outcomes and complications, ultimately leading to more tailored management strategies for this complex and vulnerable patient population.

Although patients with active cancer had significantly higher 1‐year and 2‐year mortality rates compared to those without cancer, other outcomes such as in‐hospital mortality, 30‐day mortality, in‐hospital AKI, PPM implantation, major bleeding, and in‐hospital stroke were not significantly different between the two groups. These findings suggest that TAVR could be a viable option to delay or even prevent surgery in active cancer cases. Given the comparable short‐term outcomes, it is important that these patients are not left untreated from the beginning. With advancements in cancer treatments, life expectancy may improve, and the prognosis for these patients could be extended. Therefore, TAVR may provide a significant benefit in managing severe aortic stenosis in cancer patients, offering a less invasive alternative to surgery while addressing their complex health needs.

### 5.1 Strength and Limitations

The strengths of this study include its robust methodology, comprehensive synthesis of data from 13 studies, and rigorous sensitivity analysis to ensure the accuracy of our findings. Furthermore, our approach provides clinically useful insights by focusing on patients with active malignancy, a population distinct from those with previous or cured malignancies.

There are several limitations that need to be mentioned. First, there was heterogeneity in cancer type, stage, and treatment across the studies included, which may affect the outcomes. Second, since our meta‐analysis was based on published data, we could not control for each patient's individual characteristics or include complex clinical information. Third, although statistically insignificant, publication bias cannot be entirely ruled out. In addition, since cancer patients are often excluded from trials, the included studies are primarily observational, which may introduce notable biases related to selection and reporting. Furthermore, there is a lack of long‐term follow‐up data beyond 2 years, which limits our ability to fully assess the durability of TAVR outcomes. Finally, candidates for TAVR should generally have at least 1 year of expected survival. Even in cancer patients, future studies should clearly define how they assess this issue, as it was not sufficiently addressed in the included studies.

## 6 Conclusion

The comparable short‐term outcomes of patients with active malignancy to those of non‐cancer patients make TAVR a promising treatment modality for those with severe AS. However, the higher rate of long‐term mortality underscores the importance of a combined cardiovascular and oncologic approach to optimize patient management and selection. Future studies should focus on defining how cancer type, stage, and treatment impact TAVR outcomes and explore strategies to improve long‐term survival in this vulnerable population, optimizing both cardiovascular and oncology care without jeopardizing either.

## Author Contributions

Concept development (conceived the research idea): E.A.S., S.S.N., P.S., and A.N. Study design (developed the methodology for generating results): M.H.K., R.C., A.G., and S.S.N. Supervision (provided oversight, managed the organization, execution, and manuscript writing): D.A., M.H., and E.A.S. Data collection/processing (conducted experiments, managed patient data, organized, and reported the data): M.H., A.G., P.S., N.R.K., and M.H.K. Data analysis/interpretation (performed statistical analysis, evaluation, and result presentation): E.A.S., A.G., and A.N. Literature search (performed the review of the literature): A.L.N., N.R.K., and D.A. Manuscript drafting (wrote substantial portions of the manuscript): all authors.

## Ethics Statement

The authors have nothing to report.

## Consent

The authors have nothing to report.

## Conflicts of Interest

The authors declare no conflicts of interest.

## Supporting information

Supporting information.

## Data Availability

The data that support the findings of this study are available from the corresponding author upon reasonable request.
