# Supplementary material for: Clinical Outcomes and Prognostic Implications of TAVR in Patients With Active Cancer: A Meta‐Analysis
Source: Clin Cardiol. 2025 Mar 24;48(3):e70121. doi: 10.1002/clc.70121 (PMC11931327; doi:10.1002/clc.70121)
Supplement: Supplementary file 1 — Supporting information. [file CLC-48-e70121-s001.docx]

**Table S1**: Search strategy of included studies

| Database | Search Formula |
| --- | --- |
| PubMed | ("Transcatheter Aortic Valve Replacement"[Mesh] OR "Transcatheter Aortic Valve Replacement" OR "Transcatheter Aortic Valve Implantation" OR TAVR OR TAVI) AND ("Neoplasms"[Mesh] OR cancer OR neoplasm* OR carcinoma* OR malignan* OR oncolog* OR tumor* OR tumour*) |
| Web of Science | (("Transcatheter Aortic Valve Replacement" OR "Transcatheter Aortic Valve Replacement" OR "Transcatheter Aortic Valve Implantation" OR "TAVR" OR "TAVI") AND ("Neoplasms" OR cancer OR neoplasm* OR carcinoma* OR malignan* OR oncolog* OR tumor* OR tumour*)) |
| Scopus | TITLE-ABS-KEY ( "Transcatheter Aortic Valve Replacement" OR "Transcatheter Aortic Valve Replacement" OR "Transcatheter Aortic Valve Implantation" OR "TAVR" OR "TAVI" ) AND TITLE-ABS-KEY ( "Neoplasms" OR cancer OR neoplasm* OR carcinoma* OR malignan* OR oncolog* OR tumor* OR tumour* ) |

**
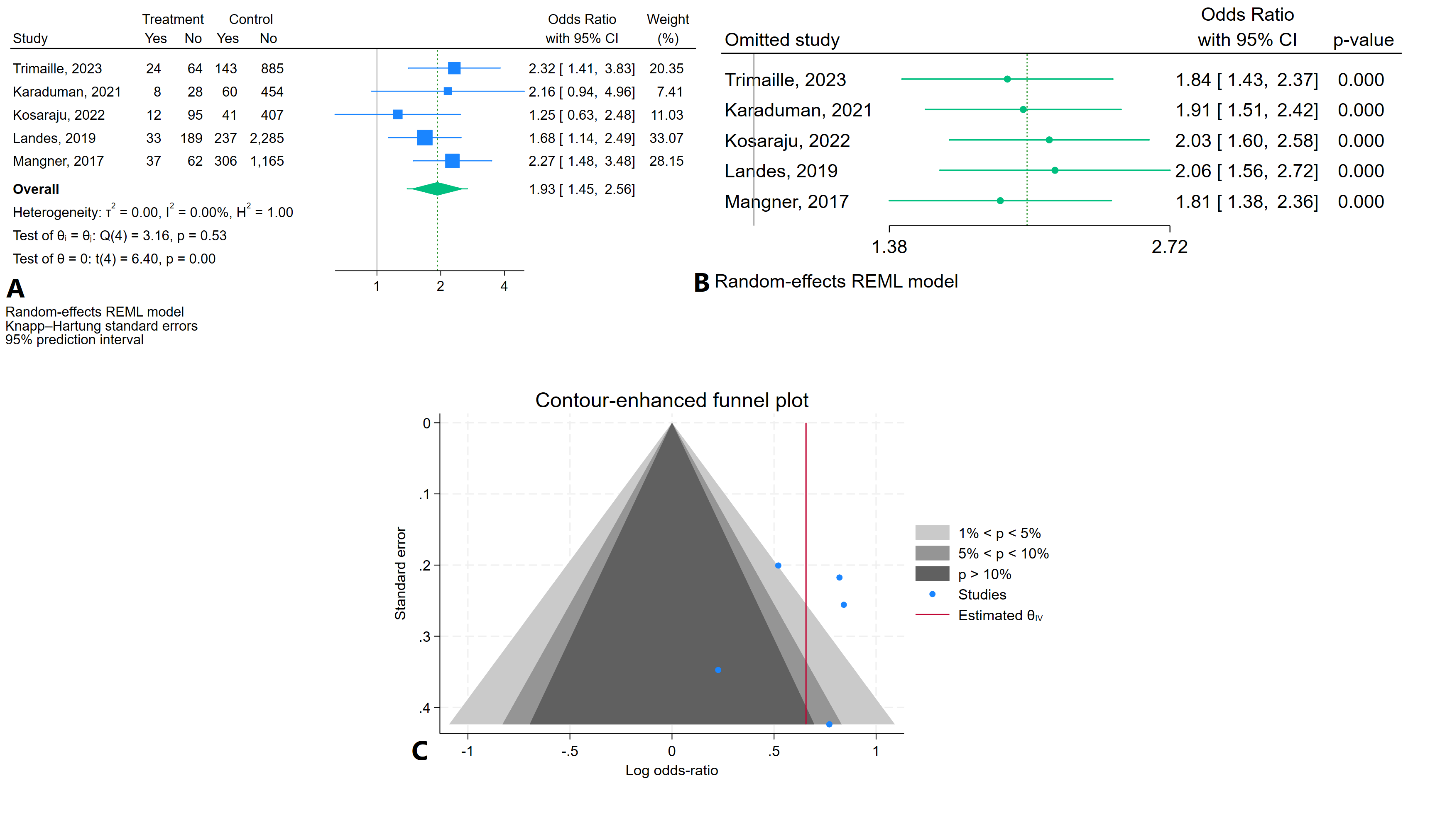
 Figure** **S1:** One-year mortality in patients with active cancer compared to healthy individuals (A) Forest plot (B) Sensitivity analysis (C) Contour-enhanced funnel plot

**
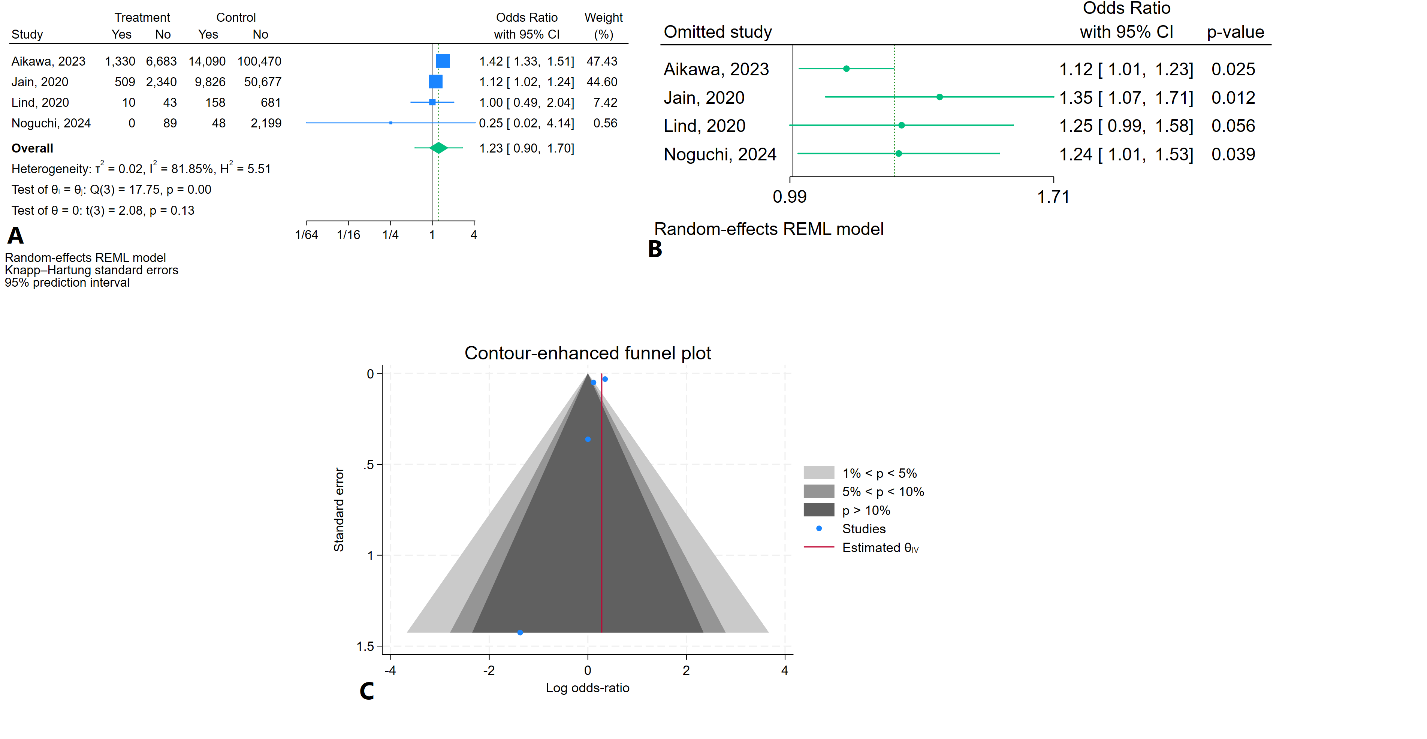
**

**Figure S2:** In-hospital AKI in patients with active cancer compared to healthy individuals (A) Forest plot (B) Sensitivity analysis (C) Contour-enhanced funnel plot

**
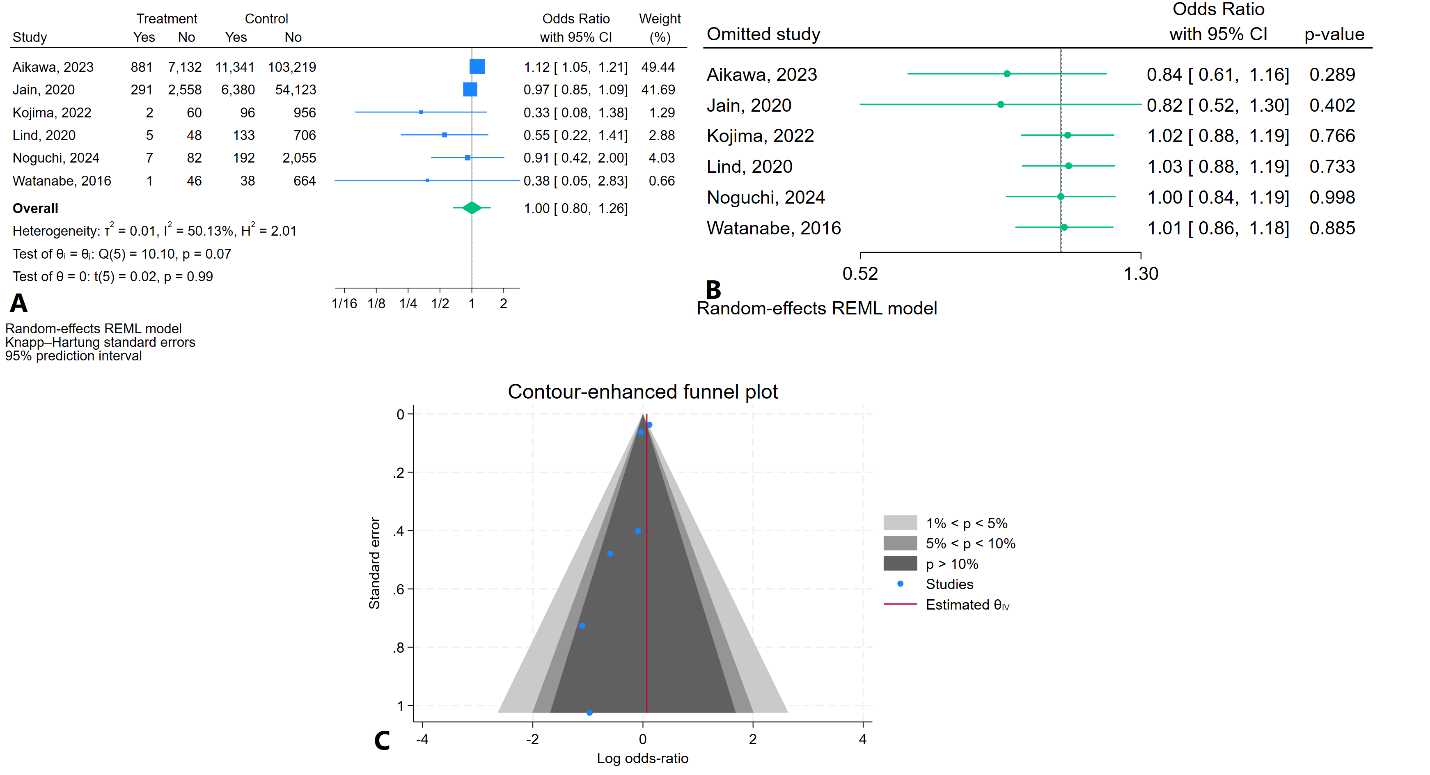
**

**Figure S3:** PPM implantation in patients with active cancer compared to healthy individuals (A) Forest plot (B) Sensitivity analysis (C) Contour-enhanced funnel plot


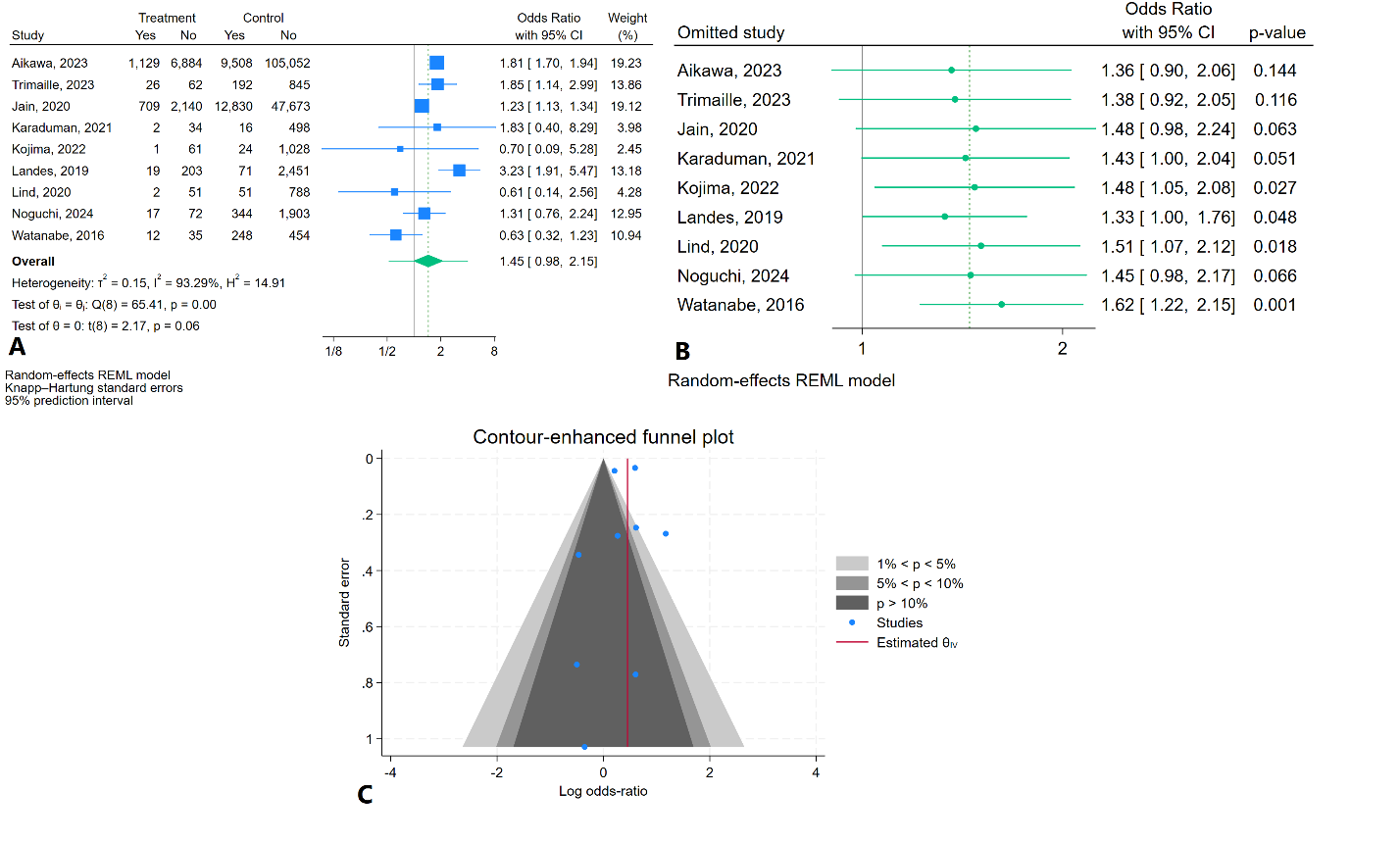


**Figure S4:** Major bleeding in patients with active cancer compared to healthy individuals (A) Forest plot (B) Sensitivity analysis (C) Contour-enhanced funnel plot


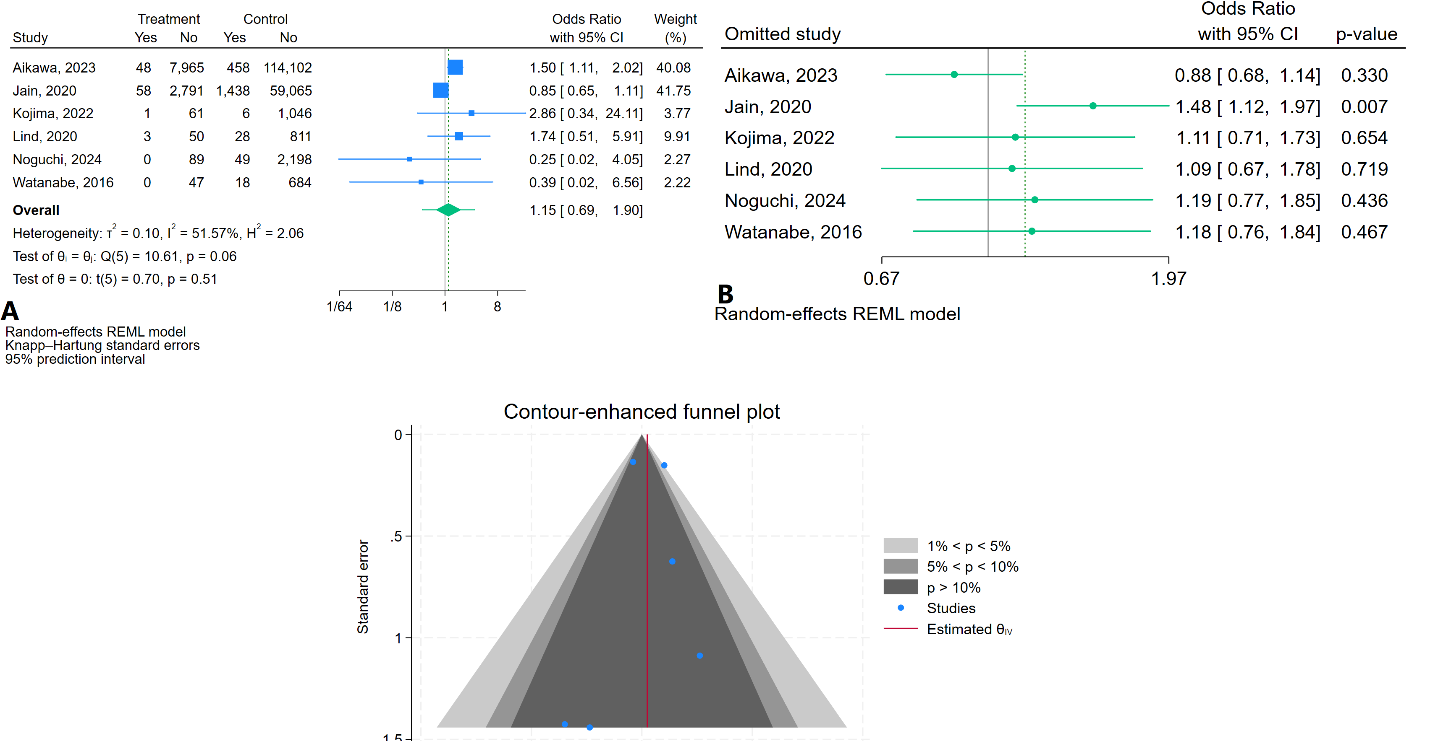


**Figure S5:** In-hospital stroke in patients with active cancer compared to healthy individuals (A) Forest plot (B) Sensitivity analysis (C) Contour-enhanced funnel plot

**
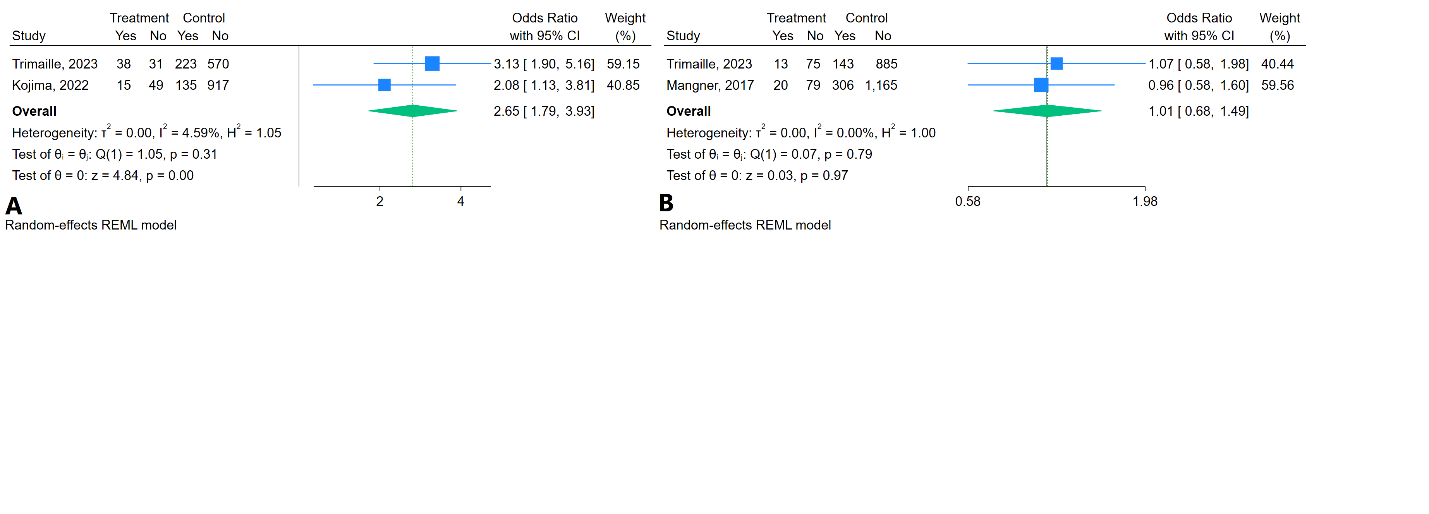
Figure S6:** Forest plots of two-year mortality (A) and one-year cardiovascular disease (B) in patients with active cancer compared to healthy individuals
